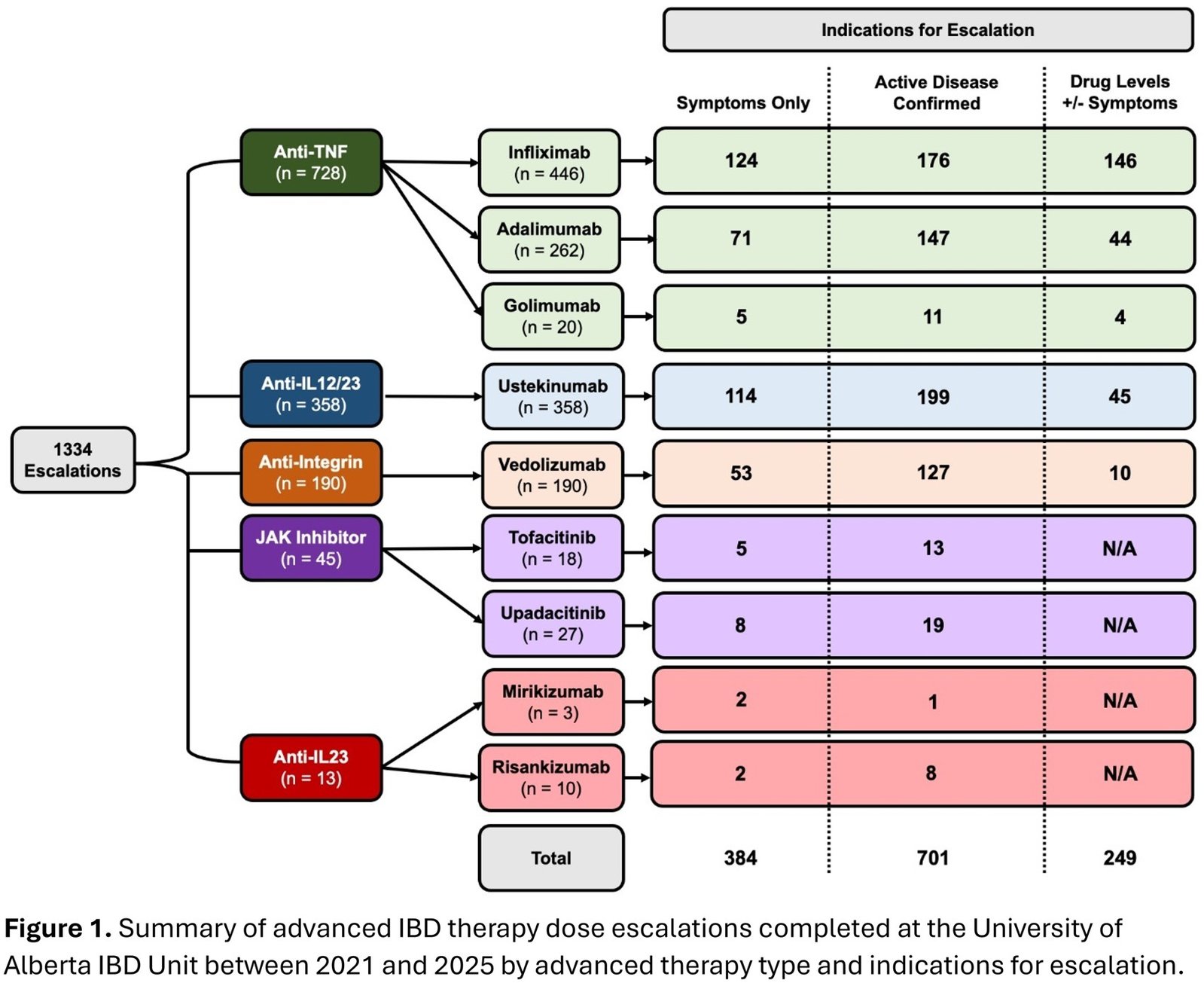# Poster Session II - A215 OVER 25% OF INFLAMMATORY BOWEL DISEASE ADVANCED THERAPY DOSE ESCALATIONS ARE BASED ON SYMPTOMS ALONE: A QUALITY IMPROVEMENT OPPORTUNITY

**DOI:** 10.1093/jcag/gwaf042.215

**Published:** 2026-02-13

**Authors:** S A MacKay, C McCabe-Woodrow, K O’Hara-Banack, P Eliuk, K Kroeker

**Affiliations:** University of Alberta Division of Gastroenterology, Edmonton, AB, Canada; University of Alberta Hospital, Edmonton, AB, Canada; University of Alberta Hospital, Edmonton, AB, Canada; University of Alberta Hospital, Edmonton, AB, Canada; University of Alberta Division of Gastroenterology, Edmonton, AB, Canada

## Abstract

**Background:**

Choosing Wisely Canada (CWC) recommends that long-term therapies for Inflammatory Bowel Disease (IBD) should not be initiated or escalated based only on symptoms. Dose escalations of advanced IBD therapies can lead to increased costs, potential safety concerns, and may subject patients to long-term use of higher than necessary advanced therapy doses. This study provides a single-centre examination of advanced IBD therapy dose escalations including what indications were present to inform these management changes.

**Aims:**

To assess the proportion of advanced IBD therapy changes made between 2021 and 2025 at the University of Alberta IBD Unit which followed current CWC recommendations.

**Methods:**

This retrospective cohort quality improvement study reviewed advanced IBD therapy dose escalations to determine which reported indications were present at time of escalation. Possible indications included patient symptoms, abnormal biomarkers (i.e., fecal calprotectin [FCP], C-reactive protein), active disease on endoscopy, imaging results (i.e., intestinal ultrasound, CT and/or MR enterography), serum drug and anti-drug antibody levels, and missed doses. Descriptive statistics were calculated for the overall cohort and subgroups for each advanced therapy agent.

**Results:**

In total, 1334 advanced IBD therapy dose escalations for 1081 patients were reviewed. 384 (28.8%) dose escalations were made based on patient symptoms alone and 701 (52.5%) were made in response to active disease being identified. The remaining 249 (18.7%) escalations were based on serum drug and anti-drug antibodies levels, with or without concurrent symptoms. Anti-TNF agents were the most frequently escalated medication class with 728 (54.6%) escalations, followed by anti-IL12/23 with 358 (26.8%), anti-integrins with 190 (14.2%), JAK inhibitors with 45 (3.4%), and anti-IL23 with 13 (1.0%). Evidence of disease activity was most commonly obtained from FCP levels (48.4%) and endoscopic findings (47.4%). Dose de-escalations were also identified, with 54 de-escalations documented between 2021 and 2025.

**Conclusions:**

This study demonstrates the need for ongoing improvement in determining active IBD prior to advanced therapy dose escalations. Recommendations regarding the use of serum drug and anti-drug antibody levels to inform dose escalations could potentially minimize the discordance between current CWC recommendations and clinical practice as shown in this study. Further research assessing the clinical outcomes of patients who were dose escalated on the basis of symptoms alone is required.

**Funding Agencies:**

None